# Improving Estimation of Fiber Orientations in Diffusion MRI Using Inter-Subject Information Sharing

**DOI:** 10.1038/srep37847

**Published:** 2016-11-28

**Authors:** Geng Chen, Pei Zhang, Ke Li, Chong-Yaw Wee, Yafeng Wu, Dinggang Shen, Pew-Thian Yap

**Affiliations:** 1 Data Processing Center, Northwestern Polytechnical University, Xi’an, 712000, China; 2 Department of Radiology and Biomedical Research Imaging Center (BRIC), University of North Carolina at Chapel Hill, NC, 27599, USA; 3 Fundamental Science on Ergonomics and Environment Control Laboratory, Beihang University, Beijing, 100191, China; 4 Department of Brain and Cognitive Engineering, Korea University, Seoul, 02841, Korea

## Abstract

Diffusion magnetic resonance imaging is widely used to investigate diffusion patterns of water molecules in the human brain. It provides information that is useful for tracing axonal bundles and inferring brain connectivity. Diffusion axonal tracing, namely tractography, relies on local directional information provided by the orientation distribution functions (ODFs) estimated at each voxel. To accurately estimate ODFs, data of good signal-to-noise ratio and sufficient angular samples are desired. This is however not always available in practice. In this paper, we propose to improve ODF estimation by using inter-subject image correlation. Specifically, we demonstrate that diffusion-weighted images acquired from different subjects can be transformed to the space of a target subject to drastically increase the number of angular samples to improve ODF estimation. This is largely due to the incoherence of the angular samples generated when the diffusion signals are reoriented and warped to the target space. To reorient the diffusion signals, we propose a new spatial normalization method that directly acts on diffusion signals using local affine transforms. Experiments on both synthetic data and real data show that our method can reduce noise-induced artifacts, such as spurious ODF peaks, and yield more coherent orientations.

Diffusion magnetic resonance imaging (MRI)^1^ provides information on brain circuitry by observing the diffusion patterns of water molecules in the human brain. To trace brain connections^2^, diffusion tractography algorithms rely on information provided by local fiber orientations, which are often represented by a quantity called the orientation distribution function (ODF). The white matter pathways estimated by tractography provide valuable information for neuroscience studies investigating human brain development, aging, and disorders^2^,^3^,^4^,^5^,^6^,^7^. Tractography also provides surgeons with valuable information for surgical planning^8^. Accurate ODF estimation is key to successful tractography. Two major factors affect the estimation accuracy of ODFs: (1) The number of diffusion-sensitizing gradient directions used to acquire the diffusion data and (2) The signal-to-noise ratio (SNR) of the data. Figure 1 shows that ODF estimation improves when a sufficient number of gradient directions are used (top row) and gets worse with heavy noise (bottom row).

Varentsova *et al*.^9^ introduced a post-processing approach to increase the number of gradient directions for improving ODF estimation in an atlas. The key idea is to make use of the orientation incoherence of the diffusion signals when they are reoriented and warped to a common space. This incoherence is a direct result of the variation of brain shape and the position of the head when scanned. A major drawback of this approach is that only rotation is considered when reorienting the diffusion signals. We show that this deteriorates ODF estimation when transformations such as shearing are involved. This approach is also limited due to its implicit assumption that the images are perfectly aligned after spatial registration. This assumption almost never holds in the real-world scenario and will cause blurring of structures that are misaligned.

A number of methods for denoising the diffusion MRI data have been proposed^10^,^11^,^12^,^13^,^14^,^15^. These methods are effective for enhancing the signal SNR, but to improve the ODF estimation, removing noise is not sufficient – another important aspect is to enhance angular resolution. In this paper we seek to better estimate ODFs by concurrent edge-preserving signal denoising and angular resolution enhancement. Signal denoising is carried out in a way similar to non-local means (NLM)^16^. NLM utilizes block matching to gather image self-similarity information and then performs weighted averaging to remove the noise. However, unlike the conventional NLM, our method leverages both self and inter-subject similarity. The underlying assumption is that the possibility of finding repeating structures from a collection of scans of different individuals is higher than a single scan from the same individual. In transferring information from images of multiple individuals to the space of the target individual for denoising, we make available signals from incoherent gradient directions for improving ODF estimation. This is illustrated in Fig. 2, where we show that the effective number of gradient directions can be significantly increased by inter-subject information transfer. For this purpose, we propose a signal reorientation method that utilizes the full affine transform estimated locally from a non-linear deformation field. Our method differs from that of Varentsova *et al*.^9^, which only uses the rotation component of the affine transform. Moreover, inter-subject misalignment, which is not taken into consideration in their work, is solved in our framework by block matching to mitigate the influence of mismatching structures. Finally, we integrate both block matching based denoising and angular resolution enhancement into a unified framework to improve ODF estimation. Our method is based on the assumption that the images are collected using a common imaging protocol, allowing information to be shared among subjects. This assumption is not particularly restrictive because in most studies images are typically scanned for a cohort of subjects with a common acquisition protocol. Part of this work has been reported in our recent workshop paper^17^. Herein, we provide additional examples, results, derivations, and insights that are not part of the workshop publication.

## Results

### Datasets

#### Synthetic dataset

A set of single pixel images were generated to evaluate the performance of our method in reconstructing ODFs from low angular resolution noisy data. Both single-direction and two-direction cases were considered. For the latter, the angular separation between two directions was set to 45°, 60° and 90°. Eight ground truth images for these two cases were generated using 6 and 21 gradient directions. Ten reoriented images were generated for each ground truth image by applying affine transformations to the principal directions of the tensors. The affine transformations include random rotation ([−90°, 90°]) around the axis perpendicular to the image plane and shearing ([−0.5, 0.5]) within the image plane. Four levels of Rician noise (3%, 5%, 7% and 9%) were added to the ground truth image and the reoriented images. The noise-perturbed ground truth image was used as the target image and the noisy reoriented images were the reference images.

#### Real dataset

The real dataset consists of diffusion-weighted (DW) images from 9 subjects. One subject was used as the target and the other subjects as references. All images were acquired using a Siemens 3T TRIO MR scanner following a standard imaging protocol: 30 diffusion directions isotropically distributed on a hemisphere, *b* = 1,000 s/mm^2^, one image with no diffusion weighting, 128 × 128 imaging matrix, voxel size of 2 × 2 × 2 × mm^3^, TE = 81 ms, TR = 7,618 ms, 1 average. Informed written consent was obtained from the subjects and the experimental protocols were approved by the Institutional Review Board of the University of North Carolina (UNC) School of Medicine. The study was carried out in accordance with the approved guidelines.

### Experimental setting

For the real dataset, the reference DW images were registered to the target space by diffeomorphic demons^18^ using the reference and target fractional anisotropy (FA) images. Based on the estimated deformation field, the reference DW images were warped to the target space using DW spatial warping^19^. The warped reference DW images were then used for multi-channel block matching with respect to the target DW images. Note that we performed block matching on the warped DW images instead of scalar images, such as those based on FA, or the non-diffusion-weighted image, resulting in more accurate matching of fiber orientations. For the synthetic dataset, block matching was ignored and reorientation was performed based on the affine matrix.

For quantitative evaluation, the Orientational Discrepancy (OD) metric^20^ was used. OD is a measure of the angular difference between two sets of directions. For OD calculation, the directions of the ODF peaks were detected and stored^19^. Let **G**_T_(**x**) be the set of directions at location **x** in the ground truth image and **G**_S_(**x**) be the set of corresponding directions in a comparison image. The OD is defined as


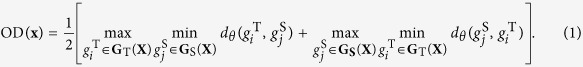


The above equation consists of two symmetrical parts that represent both the points of view of the ground truth peaks and the comparison peaks. For example, the first part of (2) indicates the maximum angle discrepancy between two sets of directions as seen from the ground truth point of view:


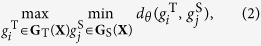


where 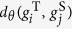 gives the angle difference between 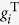 and 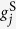, i.e.,


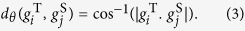


We also performed the evaluation based on fiber tracts. For this purpose, we use streamline tractography^21^,^22^ to generate the fiber tracts. The number of detected peak directions of each ODF was limited to three. The voxels with FA values larger than 0.4 were selected as seeds. The stopping FA value was set to 0.2 and the maximum allowed turning angle is set to 60°.

### Reorientation evaluation

**Figure 3:**
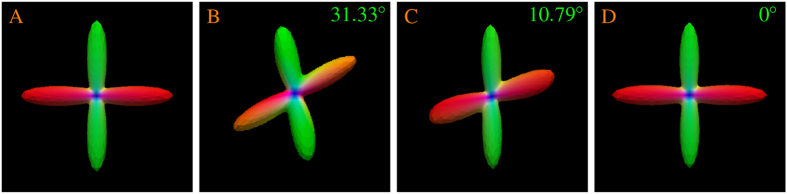
Reorientation performance. ODF estimated using (**A**) ground truth data, (**B**) test data, (**C**) rotation only, and (**D**) the proposed method.

We first demonstrate that our reorientation method is producing correct results. Figure 3 indicates that the ODF estimated from the reoriented data is very close to the ground truth. In the figure, the OD values shown in the right corners confirms that the ODF peaks given by our method exactly matches those of the ground truth, whereas the rotation-only method^9^ results in an OD value of 10.79°. Both rotation and shearing were used to generate the test data.

### Synthetic data experiment

**Figure 4:**
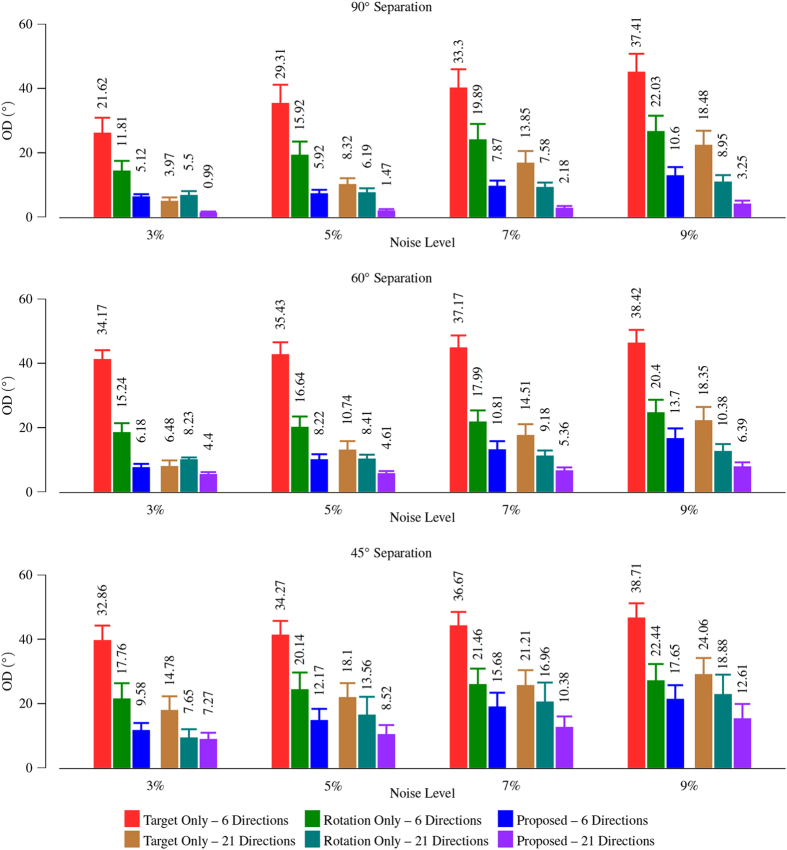
Average OD comparison using two-direction synthetic data. Three cases were compared: (1) Using only the target image; (2) Using the proposed method; and (3) Using the proposed method but only rotation was used for reorientation. Four noise levels and two sets of gradient directions were involved. The error bars indicate the standard deviations. For the proposed method, 10 reference images were used.

We repeatedly generated the synthetic data and ran the experiment 900 times. The mean and standard deviation of OD values were reported. Figure 4 shows that our method significantly reduces the mean OD on the two-direction crossing synthetic data. The small mean OD indicates that the estimated peaks are close to the ground truth. Compared with the results given by using the target image only, the maximum improvement is 27.99° when the noise level is 3%. This is for the case of 6 gradient directions, where each pair of directions are separated by an angle of 60°.

**Figure 5:**
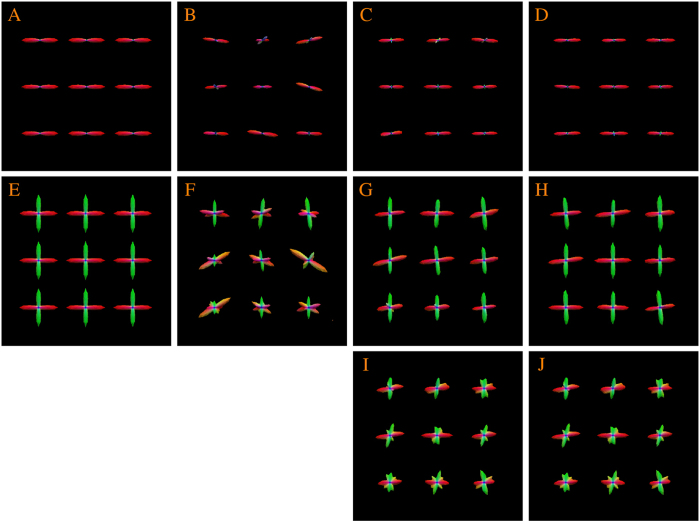
Comparison of ODFs. To view the results in a better form, we randomly picked 9 sets of single pixel results and combined them together to form a 9 × 9 ODF maps. (**A**) and (**E**) Ground truth ODFs. (**B**) and (**F**) ODFs estimated using only the target, which was generated using 5% noise and 6 gradient directions; (**C**) and (**G**) ODFs estimated using the proposed method with 5 reference images. (**D**) and (**H**) ODFs estimated using the proposed method with 10 reference images. (**I**) and (**J**) Results when only rotation was used for reorientation.

The ODF glyphs are shown for visual inspection in Fig. 5. The estimated ODF glyphs look very similar to the ground truth. We ran the same experiment by performing only rotation for reorientation, as done in Varentsova *et al*.’s method^9^. The results, shown in Fig. 5, indicate that this will cause spurious peaks that are not observed in the ground truth. The superiority of our method over the rotation-only approach is confirmed in Fig. 4.

### Real data experiment

**Figure 6:**
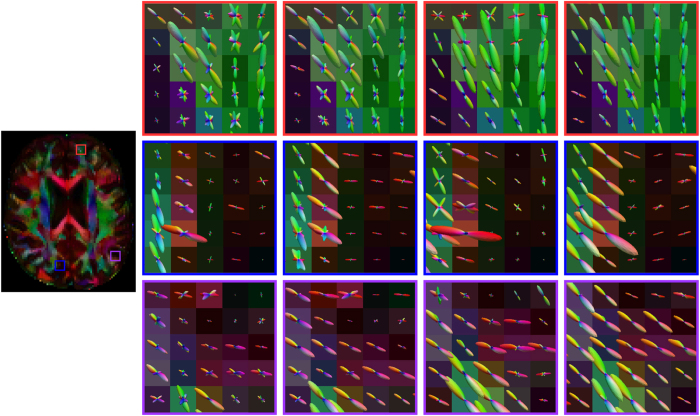
Axial view of ODFs. (Far Left) Reference FA image. (Left to right) ODFs estimated using the target dataset, NLM-denoised target dataset, the proposed method but without block matching, and the proposed method.

**Figure 7:**
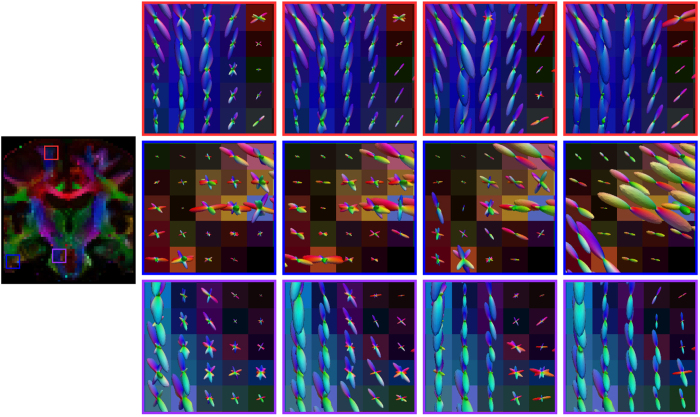
Coronal view of ODFs. Similar to Fig. 6, but in coronal view.

**Figure 8:**
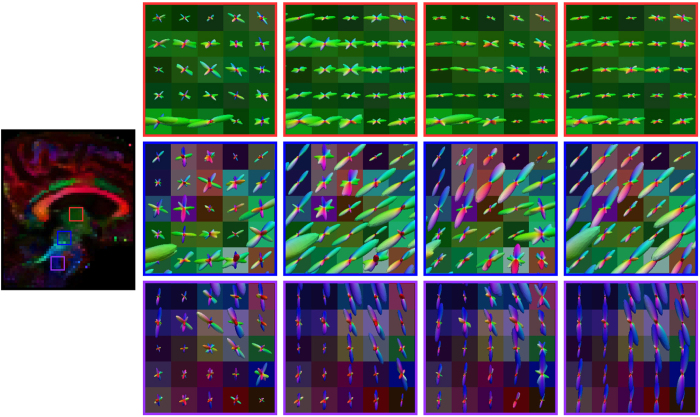
Sagittal view of ODFs. Similar to Fig. 6, but in sagittal view.

For the real data, the ODFs are shown in three views in Figs 6, 7 and 8. We show the results for the target dataset, the NLM-denoised target dataset, the proposed method without block matching, and the full implementation of the proposed method. For NLM denoising, we used a multi-spectral version of the algorithm^12^. NLM denoising was first performed on the target dataset, and the denoised dataset was then used for ODF estimation. For evaluating the effectiveness of block matching, we referred to Varentsova *et al*.’s method^9^ and disabled the block-matching component in our method so that diffusion signals at the same spatial location of all scans are used for ODF estimation. We can observe from each view that, other than the proposed method, the ODFs estimated exhibit spurious peaks The ODFs are also not as coherent as those estimated using the proposed method.

**Figure 9:**
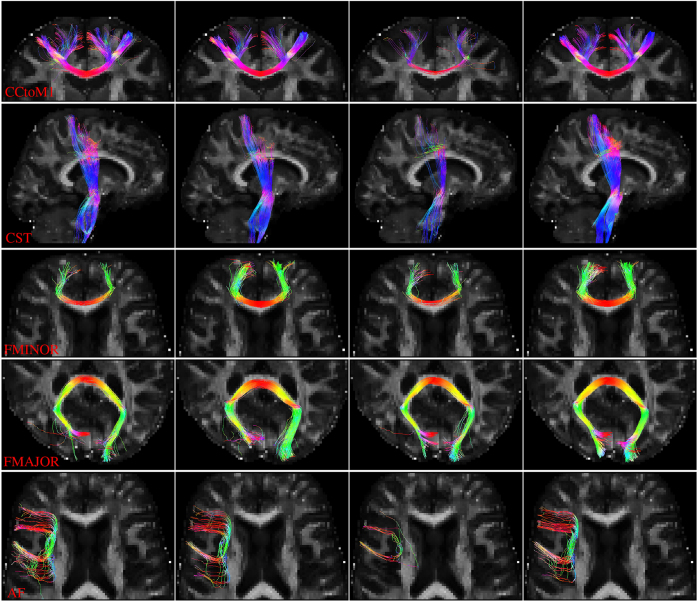
Tractography. Four representative sets of tractography results using the target dataset, NLM-denoised target dataset, the proposed method but without block matching, and the proposed method. The ROIs used in selecting target bundles are defined in Table 1.

Based on the ROIs described in Table 1, we extracted four representative tract bundles from the tracts given by whole brain tractography. The resulting tracts, shown in Fig. 9, indicate that the proposed method gives cleaner and richer fiber tracts compared with the other three methods. When block matching is not used, a significant amount of fiber tracts are missing. The proposed method gives fuller and smoother fiber tracts. Although NLM improves the quality of fiber tracts, we can still observe a lot of spurious fiber tracts resulting from inaccurate ODF estimation.

**Figure 10:**
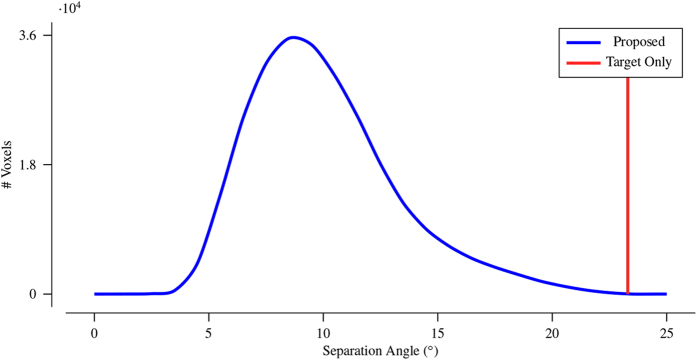
The angular-separation histogram. The angular separation of the target data is marked by the red line and is equal to 23.3°.

Figure 10 shows the histogram of the largest angular separation of the gradient directions associated with the measured DW signals. Compared with the large angular separation given by using only the target image (indicated by the red line in the figure), the angular separation is significantly reduced by the proposed method. This result in greater angular resolution and hence improves ODF estimation and tractography.

**Figure 11:**
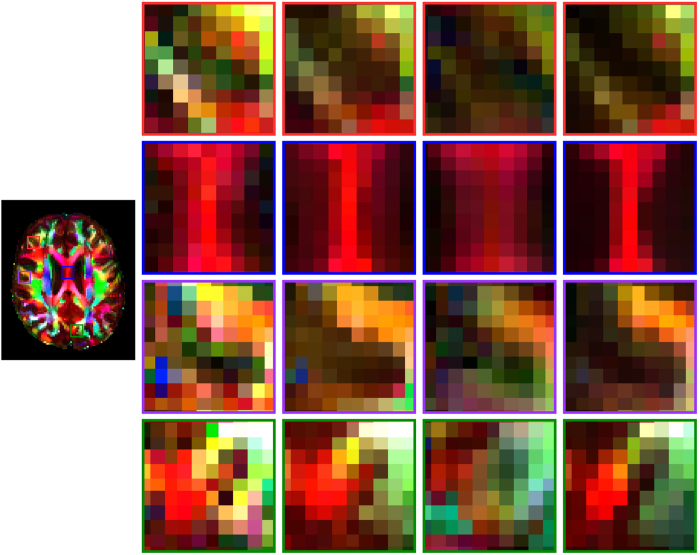
Colored FA. Direction-encoded color FA images given by the target dataset, NLM-denoised target dataset, the proposed method without block matching, and the proposed method.

Finally, the colored FA images, shown in Fig. 11, confirm the advantages of the proposed method. Sharp and clean FA image was obtained by our method. In contrast, when block matching in not used, as in Varentsova *et al*.’s method^9^, plenty of fuzzy structures were introduced. At locations affected by registration errors, the reference diffusion signals often differ significantly from the target diffusion signals, therefore causing fuzziness. This can be overcome by block matching.

## Discussion

Although inspired by Varentsova *et al*.’s work^9^, our method presents important distinctions and overcomes some crucial drawbacks in their method. Similar to Varentsova *et al*.’s work^9^, samples from incoherent directions from different subjects are used to enhance angular resolution. However, dissimilar to Varentsova *et al*.’s work^9^, which aims to construct a diffusion atlas, the goal of our work is to improve ODF estimation based on the diffusion data of a single subject by borrowing information from other subjects.

Our method leverages block matching to mitigate registration error and to gather redundant information for improved ODF estimation. In practice, reference images cannot be perfectly aligned to the target image due to potential registration error. If information is transferred directly from the reference images to the target image without consideration of misalignment errors, as done in Varentsova *et al*.’s work^9^, plenty of artifacts due to mismatched structures will be introduced. As shown in Figs 6, 7, 8 and 9, spurious ODF peaks and fibers can be observed when block matching is not used.

In Varentsova *et al*.’s work^9^, only rotation is taken into account when the diffusion signals are reoriented. As shown in Figs 3 and 5, the rotation-only approach fails to consider factors such as shearing. We proposed a reorientation procedure that gives the following advantages: (1) Full affine transform is applied for reorientation. (2) Our method does not require fitting any model to the data, unlike some model-based methods^23^,^24^. (3) Our method performs reorientation based directly on the gradient directions, instead of ODF^24^, facilitating transferring of incoherent angular information to the target image. Better results are given by the proposed method as shown in Figs 4 and 5.

Our method is designed to work on a group of diffusion data acquired using the same imaging protocol. This basic requirement allows information to be shared across different subjects. This requirement can be relaxed by extending the method to work with different protocols. This implies that an even greater number of images can be used for further improving estimation. This can be achieved by first performing inter-protocol data harmonization^25^,^26^. Correction of the heterogeneity among images acquire using different protocols makes information sharing between them feasible.

In summary, we have proposed a method for improving ODF estimation by borrowing information across subjects. Information is borrowed from multiple datasets to simultaneously remove noise and to enhance angular resolution. For information transfer between datasets, we proposed a reorientation method that directly acts on diffusion signals using the full affine transform. Extensive experiments on both synthetic and real data show improved ODF estimation, despite using noisy data with insufficient angular sampling. Further tractography-based validation demonstrates that our approach produces fiber tracts that are cleaner and smoother.

## Method

### Overview

**Figure 12:**
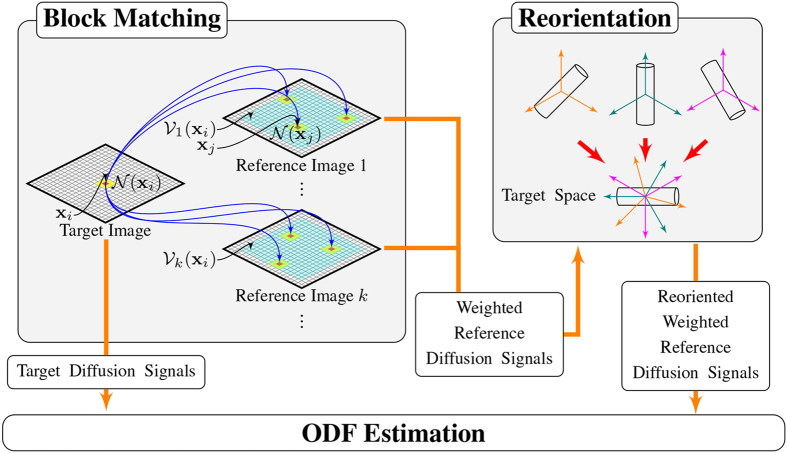
Overview. Three components of our method: (1) Block matching for identifying corresponding voxels from the reference images, (2) Reorientation of the reference diffusion signals, and (3) ODF estimation.

Suppose we have a group of reference images acquired from different individuals (possibly also including the target individual), the goal is to improve ODF estimation for the target image with the help of the reference images. This is achieved in three steps: (1) Block matching, (2) Reorientation, and (3) ODF estimation. Each step is detailed below. See Fig. 12 for an overview.

### Block matching

We first warp all the reference images to the target space. For each voxel in the target image, we then determine the matching voxels in the reference images via robust block matching, similar to that used in NLM^16^. A similarity weight is determined for each matching voxel and will be used for ODF estimation. NLM relies on repeating structures in an image. However, this might be challenging due to the complex anatomy of the human brain and fine unique structures might not find matching candidates. To address this issue, we extend NLM by performing block matching across images, significantly increasing the chance of finding similar structures. Gross misalignment between images is first dealt with using non-linear registration and residual misalignment is then overcome using block matching.

Let 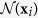 be a 3D block neighbourhood centered at 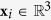. The size of 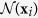 is (2*d* + 1)^3^, where *d* is the neighborhood radius. Let 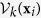 be the search volume centered at **x**_***i***_ in reference image *k*. The size of 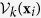 is (2*m* + 1)^3^, where *m* is the search radius. Let **u(x**_*i*_) be the intensity value at **x**_*i*_ and 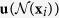 be a vector that represents the intensity values of all voxels within 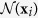. The unnormalized weight, indicating similarity between the neighborhoods of a voxel, **x**_*i*_, in the target image, and a voxel, 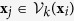, in the reference image is computed as


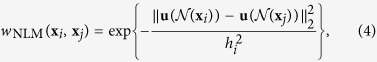


where *h*_*i*_ controls the attenuation of the exponential function. Coupé *et al*.^27^ suggested to set 
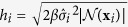
, where 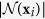 is the cardinality of 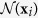, *β* is a constant that is set to 1, 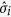 is an estimate of the standard deviation of the noise at **x**_*i*_, which is spatial-adaptively estimated^28^. In our case, we set block radius *d* = 1 voxel and search radius *m* = 2 voxels. Note that the search range of 5 × 5 × 5 is sufficient for the inter-subject mismatching correction due to the pre-registration of the DW images^29^.

For each voxel in the target image, block matching leads to a set of corresponding voxels and associated similarity weights in the reference images. Specifically, given **x**_*i*_ in the target, we have 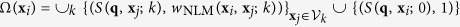, where *S*(**q**, **x**; *k*), *k* > 0 is the diffusion-attenuated signal collected at **x**_*j*_ with wavevector **q** in the *k*-th reference dataset, and *S*(**q**, **x**_*i*_; 0) is the signal measured in the target dataset.

### Reorientation

The diffusion signal *S*(**q**, **x**_*j*_) in Ω(**x**_*i*_) has to be reoriented before it can be used for ODF estimation. A number of methods have been proposed for reorientation of diffusion data^23^,^24^,^30^,^31^,^32^. However, these methods perform reorientation on the ODFs rather than diffusion signals. That is, a diffusion model is first fitted to the data to estimate the ODFs, which are then reorientated according to locally estimated affine transforms. For the purpose of this work, we propose a new method for direct reorientation of the diffusion signals. Unlike Varentsova *et al*.’s reorientation method^9^, which only uses the rotation component of the affine transform, our method makes full use of the affine transform and hence results in more accurate results. The MR signal attenuation is defined as *E*(**q**, **x**_*j*_) = *S*(**q**, **x**_*j*_)/*S*_0_(**x**_*j*_), where *S*_0_(**x**_*j*_) is the base signal without diffusion-sensitizing gradient. Then, the ODF 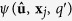, contributed by the sampling shell with radius *q*′ in *q*-space can be computed as ref. 33


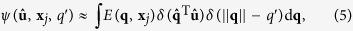


where ||·|| denotes the 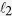 norm, 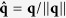, 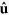 is a unit vector that represents a spatial direction, and *δ*(·) is the Dirac delta function.

We note that the deformation field for warping a moving image to a fixed image is defined in the space of the fixed image. Hence, based on the observation that the integral of ODF must be maintained after transformation, we apply a local affine matrix **A**^−1^(**x**_*j*_) computed at **x**_*j*_ to 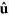 and have


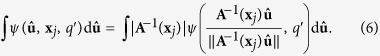


Performing change of variable 
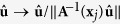
, (6) becomes


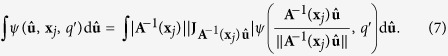


where 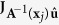 is a transformation associated with the change of variable, and |·| denotes the determinant. We then apply **A**^−1^(**x**_*j*_) to 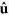 on both sides of (5) and simplify the equation to


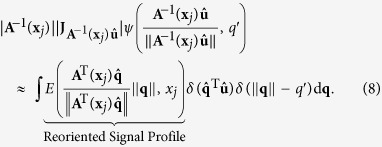


See supplementary materials for the derivation of (8). If we let


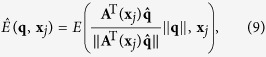


or equivalently


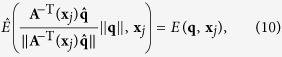


we can see that the reorientation involves transforming the signal measured at **q**, i.e., *E*(**q**, **x**_*j*_) to 
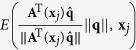
. The reoriented signal is hence 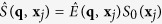. We denote the reoriented version of **Ω**(**x**_*i*_) using 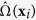.

### ODF estimation

To estimate the ODF at **x**_*i*_, 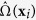 is decomposed into a linear combination of diffusion basis functions (DBFs)^34^. Dropping **x**_*i*_ for simplicity, the decomposition is given by


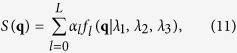


where *α*_*l*_ is the volume fraction associated with the *l*-th tensor DBF *f*_*l*_(·) and {*λ*_1_, *λ*_2_, *λ*_3_} are the three eigenvalues of the tensor. The DBF is defined as 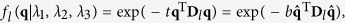 where **D**_*l*_ is a tensor defined by {*λ*_1_, *λ*_2_, *λ*_3_} and principal diffusion direction **μ**_*l*_, *t* is the diffusion time, and *b* is the diffusion weighting. For 1 ≤ *l* ≤ *L*, the tensors are anisotropic with principal diffusion directions distributed uniformly on a unit sphere. For *j* = 0, the tensor is isotropic to model free water diffusion. In practical, we estimated the diffusivities of the anisotropic tensors *λ*_1_, *λ*_2_, *λ*_3_ from the corpus callosum. Those of the isotropic tensor were estimated from the ventricles. A total of 321 orientations, generated by subdividing the faces of an icosahedron three times and discarding antipodal symmetric directions, were used as the principal diffusion directions of the DBFs.

By representing each element of set 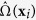 as (**s**_*n*_, *w*_*n*_) and each DBF as a column of matrix **F**_*n*_, we can solve for the volume fraction vector ***α*** = [*α*_0_, ..., *α*_L_]^T^ using 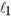-penalized weighted least-squares^19^:


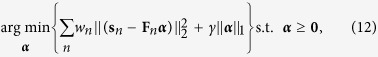


where ǁ·ǁ is the 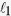-norm and *γ* ≥ 0 is a tuning parameter (0.01 in our case). **F**_*n*_ is the DBF matrix corresponding to **s**_*n*_, computed based on its reoriented gradient directions. If no reorientation is applied, **F**_*n*_ is identical for all *n*. The ODF can then be computed as


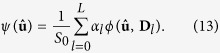


When 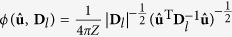, with *Z* being the normalization constant, we have the diffusion ODF^33^. When 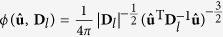, we have the constant-solid-angle diffusion ODF^35^. Finally, when 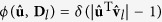, with 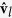 being the eigenvector of **D**_*l*_ corresponding to the largest eigenvalue, we have the fiber ODF^36^,^37^.

## Additional Information

**How to cite this article**: Chen, G. *et al*. Improving Estimation of Fiber Orientations in Diffusion MRI Using Inter-Subject Information Sharing. *Sci. Rep.* **6**, 37847; doi: 10.1038/srep37847 (2016).

**Publisher's note:** Springer Nature remains neutral with regard to jurisdictional claims in published maps and institutional affiliations.

## Supplementary Material

Supplementary Materials

## Figures and Tables

**Figure 1:**
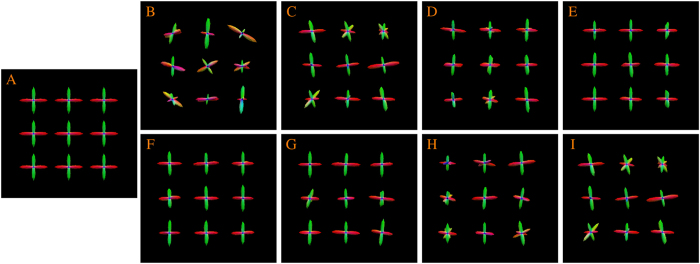
Influence of the number of gradient directions and noise on ODF estimation. (**A**) Ground truth. (**B**–**E**) ODFs estimated using 6, 21, 81, and 321 gradient directions with 9% noise. (**F**–**I**) ODFs estimated using 21 diffusion directions with 3%, 5%, 7%, and 9% noise. Gaussian noise (i.e 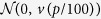) is added in the complex domain of the signal, determined by the percentage *p*, where ***v*** is the maximum signal value (150 in our case).

**Figure 2:**
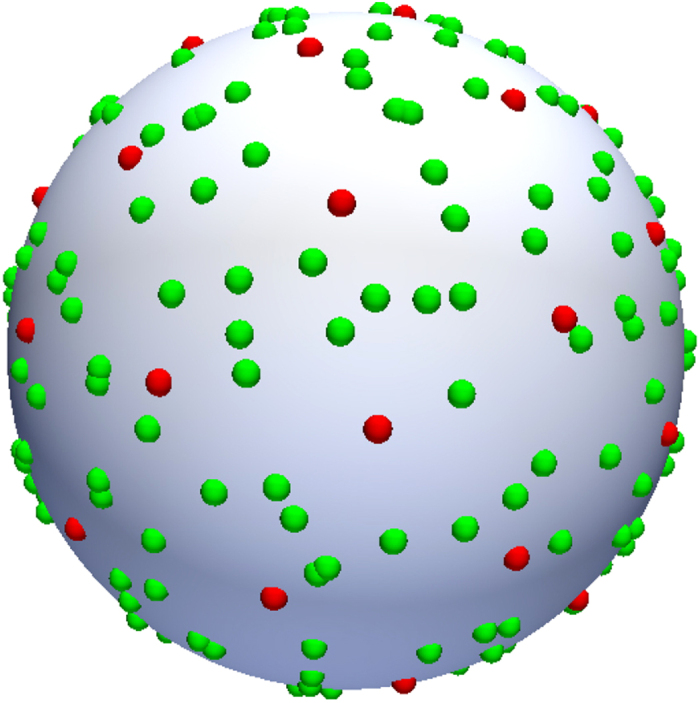
Angular resolution enhancement using inter-subject information transfer. The red points on the sphere indicate the original gradient directions. Transferring incoherent samples from 10 other scans increases the effective number of gradient directions, as indicated by the green points.

**Table 1:** ROIs used to extract fiber bundles.

Bundle	ROIs
CCtoM1	Precentral gyrus and corpus callosum
CST	Precentral gyrusPosterior limb of the internal capsule
FMAJOR	Occipital cortex and corpus callosum
FMINOR	Prefrontal cortex and corpus callosum
AF	Posterior superior temporal gyrusInferior frontal gyrus and pars opercularis
