# Supplementary material for: Improving Estimation of Fiber Orientations in Diffusion MRI Using Inter-Subject Information Sharing
Source: Sci Rep. 2016 Nov 28;6:37847. doi: 10.1038/srep37847 (PMC5124958; doi:10.1038/srep37847)
Supplement: Supplementary Materials [file srep37847-s1.pdf]

Supplementary Materials:

Improving Estimation of Fiber Orientations in Diffusion MRI Using Inter-Subject Information Sharing

(SREP-16-26694)

Geng Chen, Pei Zhang, Ke Li, Chong-Yaw Wee
Yafeng Wu, Dinggang Shen, Pew-Thian Yap

Proof for Reorientation Equation

Applying $\mathbf{A}^{-1}(\mathbf{x}_j)$ to $\hat{\mathbf{u}}$ on both sides of Eq. 5, we have

$$\begin{aligned}
&\psi\left(\frac{\mathbf{A}^{-1}(\mathbf{x}_j)\hat{\mathbf{u}}}{||\mathbf{A}^{-1}(\mathbf{x}_j)\hat{\mathbf{u}}||}, q'\right) \\
&\quad\approx \int E(\mathbf{q}, x_j)\delta\left(\hat{\mathbf{q}}^{\mathrm{T}}\frac{\mathbf{A}^{-1}(\mathbf{x}_j)\hat{\mathbf{u}}}{||\mathbf{A}^{-1}(\mathbf{x}_j)\hat{\mathbf{u}}||}\right)\delta(||\mathbf{q}||-q')d\mathbf{q} \\
&\quad= \int E(\mathbf{q}, x_j)\delta\left(\left(\mathbf{A}^{-\mathrm{T}}(\mathbf{x}_j)\hat{\mathbf{q}}\right)^{\mathrm{T}}\frac{\hat{\mathbf{u}}}{||\mathbf{A}^{-1}(\mathbf{x}_j)\hat{\mathbf{u}}||}\right)\times\delta(||\mathbf{q}||-q')d\mathbf{q}.
\end{aligned} \tag{14}$$

Performing change of variable $\frac{\mathbf{A}^{-\mathrm{T}}(\mathbf{x}_j)\hat{\mathbf{q}}}{||\mathbf{A}^{-\mathrm{T}}(\mathbf{x}_j)\hat{\mathbf{q}}||} \to \hat{\mathbf{q}}$, (14) becomes

$$\begin{aligned}
&\psi\left(\frac{\mathbf{A}^{-1}(\mathbf{x}_j)\hat{\mathbf{u}}}{||\mathbf{A}^{-1}(\mathbf{x}_j)\hat{\mathbf{u}}||}, q'\right) \\
&\quad= \int E\left(\frac{\mathbf{A}^{\mathrm{T}}(\mathbf{x}_j)\hat{\mathbf{q}}}{||\mathbf{A}^{\mathrm{T}}(\mathbf{x}_j)\hat{\mathbf{q}}||}||\mathbf{q}||, x_j\right)\delta\left(\frac{\hat{\mathbf{q}}^{\mathrm{T}}}{||\mathbf{A}^{\mathrm{T}}(\mathbf{x}_j)\hat{\mathbf{q}}||}\frac{\hat{\mathbf{u}}}{||\mathbf{A}^{-1}(\mathbf{x}_j)\hat{\mathbf{u}}||}\right) \\
&\qquad\times|\mathbf{A}^{\mathrm{T}}(\mathbf{x}_j)||\mathbf{J}_{\mathbf{A}^{\mathrm{T}}(\mathbf{x}_j)\hat{\mathbf{q}}}|\delta(||\mathbf{q}||-q')d\mathbf{q}.
\end{aligned} \tag{15}$$

Using the generalized scaling property of the Dirac-delta function [1], we have

$$\delta\left(\frac{\hat{\mathbf{q}}^{\mathrm{T}}}{||\mathbf{A}^{\mathrm{T}}(\mathbf{x}_j)\hat{\mathbf{q}}||}\frac{\hat{\mathbf{u}}}{||\mathbf{A}^{-1}(\mathbf{x}_j)\hat{\mathbf{u}}||}\right) = |\mathbf{J}_{\mathbf{A}^{\mathrm{T}}(\mathbf{x}_j)\hat{\mathbf{q}}}|^{-1}|\mathbf{J}_{\mathbf{A}^{-1}(\mathbf{x}_j)\hat{\mathbf{u}}}|^{-1}\delta\left(\hat{\mathbf{q}}^{-\mathrm{T}}\hat{\mathbf{u}}\right). \tag{16}$$

Substituting (16) in (15), we have Eq. 8.

References

[1] IM Gelfand and GE Shilov. *Generalized functions. Vol. 3, Theory of differential equations.* Academic Press, 1967.
